# Sex differences in Alzheimer’s disease: plasma MMP-9 and markers of disease severity

**DOI:** 10.1186/s13195-022-01106-4

**Published:** 2022-11-02

**Authors:** Amaryllis A. Tsiknia, Erin E. Sundermann, Emilie T. Reas, Steven D. Edland, James B. Brewer, Douglas Galasko, Sarah J. Banks, for the Alzheimer’s Disease Neuroimaging Initiative

**Affiliations:** 1 Department of Neurosciences, School of Medicine, University of California, San Diego, 9500 Gilman Dr, La Jolla, CA 92093 USA; 2 Research Service, VA San Diego Healthcare System, San Diego, CA 92161 USA; 3 Department of Psychiatry, University of California, San Diego, La Jolla, CA 92093 USA; 4 Division of Biostatistics, School of Public Health and Human Longevity Science, University of California, San Diego, La Jolla, CA 92093 USA

**Keywords:** Matrix metalloproteinase-9, Alzheimer’s disease, Mild cognitive impairment, Fluid biomarkers, Sex differences, Cognitive decline, Amyloid-β, Tau

## Abstract

**Background:**

Studies have reported higher plasma matrix metalloproteinase-9 (MMP-9) levels in mild cognitive impairment (MCI) and Alzheimer’s disease (AD). Despite evidence that MMP-9 activity and its influence on AD pathophysiology may be modulated by sex hormones, sex differences in the association between MMP-9 and AD biomarkers and cognition have not been explored.

**Methods:**

Our sample included 238 amyloid-β (Aβ)-positive participants with MCI or AD dementia from the Alzheimer’s Disease Neuroimaging Initiative (37.4% women, 74.6 ± 7.3 years). We used linear regression models to examine whether sex modified free and total plasma MMP-9 associations with CSF t-tau, p-tau181, and Aβ_42_. We used linear mixed effects models to examine whether sex modified total and free plasma MMP-9 associations with cognition, using longitudinal Mini-Mental Status Examination (MMSE) and Alzheimer’s Disease Assessment Scale-Cognitive Subscale (ADAS-cog) data.

**Results:**

Total and free MMP-9 levels did not differ by sex, but AD dementia patients had higher total MMP-9 levels than participants with MCI (*β* = 0.06 [−0.11 to −0.01], *p* = 0.031). Sex modified the association of CSF t-tau with total (*β* = 128.68 [55.37 to 201.99], *p* < 0.001) and free MMP-9 (*β* = 98.61 [33.61 to 163.62], *p* = 0.003), whereby higher total and free MMP-9 correlated with higher CSF t-tau in women and lower CSF t-tau in men. Higher free MMP-9 correlated with lower CSF p-tau181 among men (*β* = −14.98 [−27.37 to −2.58], *p* = 0.018), but not women. In participants with MCI, higher free MMP-9 levels were associated with higher CSF Aβ_42_ among men (*β* = 26.88 [4.03 to 49.73], *p* = 0.022) but not women. In the overall sample, higher free and total MMP-9 at baseline predicted worsening MMSE scores in women (*β* = −2.10 [−3.97 to −0.27], *p* = 0.027 and *β* = −2.24 [−4.32 to −0.18], *p* = 0.035) but not men. Higher free MMP-9 correlated with worse ADAS-cog scores (*β* = 12.34 [3.02 to 21.65], *p* = 0.011) in women (*β* = 12.34 [3.02 to 21.65], *p* = 0.011) but not men with AD dementia cross-sectionally but correlated with worsening ADAS-cog scores longitudinally only in men (*β* = 8.98 [0.27 to 17.68], *p* = 0.042).

**Conclusions:**

MMP-9 may have more detrimental effects on AD-related pathological and cognitive changes in women. If replicated, our findings could help uncover potential mechanisms contributing to women’s elevated susceptibility to AD.

## Background

Recent evidence suggests that vascular dysfunction may contribute to the pathogenesis and pathological progression of Alzheimer’s disease (AD) [1–3]. While several biomarkers of vascular dysfunction have been identified in relation to AD, prior studies suggest that matrix metalloproteinase-9 (MMP-9) may be particularly relevant to AD pathophysiology, due to its interaction with the apolipoprotein ε4 allele (*APOE* ε4) [4], the major genetic risk factor for sporadic AD, and its involvement in neuroinflammation [5], amyloid-β (Aβ) brain metabolism [6], and blood-brain barrier breakdown [7]. MMP-9 is a zinc-containing endopeptidase that is involved in numerous physiological processes. Its ability to degrade constituents of the extracellular matrix makes it critical to physiological processes such as angiogenesis and tissue remodeling that are essential to vascular and central nervous system health [8]. Under physiological conditions, MMP-9 activity is highly regulated by thrombospondins and tissue inhibitors of matrix metalloproteinases (TIMPs), such as TIMP-1. However, under pathological conditions that lead to overexpression, disinhibition, and/or dysregulation of MMP-9, its proteolytic activity can lead to blood-brain barrier breakdown, neuroinflammation, and demyelination [7], all of which are processes implicated in the development of AD [9]. MMP-9 can be found in both blood and in brain tissue. Peripheral sources of MMP-9 include secretion by macrophages, fibroblasts, and neurotrophils, while CNS sources of MMP-9 include production by neurons and glia and MMP-9 extravasation from blood [10–12]. Although several studies reported higher plasma MMP-9 levels and activity in mild cognitive impairment (MCI) and AD [13–16], the relationship between MMP-9 and AD pathology remains poorly understood. Prior studies in animals have shown that in the brain, MMP-9 is directly involved in the degradation and clearance of Aβ, suggesting a more protective rather than destructive role of MMP-9 in AD [6, 17, 18], while others have demonstrated that MMP-9 increases lipoprotein receptor shedding in endothelial cells, leading to reduced Aβ transport across the blood-brain barrier [19]. Additionally, evidence suggests that MMP-9 inefficiently cleaves tau, thereby releasing microtubule-binding domains of tau that facilitate its oligomerization [20, 21]. One study found that higher CSF MMP-9/TIMP-1 ratios were associated with higher levels of CSF p-tau181 and total tau (t-tau) in individuals who were at high risk of developing AD (as indicated by abnormal AD biomarkers or the presence of *APOE* ε4) and in AD dementia patients [22]. However, another group found no association between MMP-9 levels and CSF Aβ_42_, p-tau181, and t-tau [23].

Important sex differences exist in many facets of AD, evidenced in the higher prevalence of AD in women [24], its distinct course, and higher levels of pathological tau [25], with studies suggesting that these differences are particularly evident among individuals with higher levels of Aβ burden [26, 27]. A prevailing theory is that the neuroendocrine changes during menopause may predispose women to a more precipitous pathological and clinical progression following Aβ accumulation. Converging evidence suggests that in the absence of the neuro- and vaso-protective effects of estrogen, post-menopausal women may be particularly vulnerable to AD-related pathological changes [28]. Consistent with this hypothesis, several studies have demonstrated that estrogen regulates several vascular and inflammatory pathways that are relevant to the AD neuropathological cascade [28], including MMP-9 pathways. For instance, estrogen was shown to decrease MMP-9 expression and activity in human macrophages and primary cultures of rat microglia [29, 30]. Estrogen can also indirectly inhibit MMP-9 activity by inhibiting microglial activation in response to acute inflammatory stimuli, thereby reducing the production of reactive oxygen species which have been shown to activate MMP-9 [31]. Consistent with the hypothesis that estrogen influences MMP-9 expression and activity, Hu et al. found that post-menopausal women treated with either conjugated equine estrogen (CEE) alone or CEE and progestin had decreased plasma MMP-9 levels compared to untreated post-menopausal women [32]. A study of human neuroblastoma cells demonstrated that estrogen treatment increased MMP-9-mediated degradation of Aβ, suggesting that the regulatory role of estrogen in MMP-9 activity may constitute a neuroprotective mechanism against the pathological progression of AD [33]. These findings suggest that in the absence of the regulatory effects of estrogen, post-menopausal women may exhibit greater MMP-9 dysregulation and possibly exhibit worse pathological and cognitive outcomes in relation to higher plasma MMP-9 levels.

While higher MMP-9 levels have been shown to correlate with higher levels of AD biomarkers and faster cognitive decline among AD and MCI patients and individuals with genetic risk for AD [22, 23], sex differences in these associations have not been examined. Assessing a potential modulating effect of sex on the association of MMP-9 levels with markers of AD pathology and cognitive decline will help us better understand the factors influencing whether its role in AD is neuroprotective or neurotoxic. Therefore, the aim of the present study was to determine whether the association between plasma MMP-9 and CSF AD biomarker levels and cognitive decline is modulated by sex in individuals with abnormal levels of Aβ pathology. We hypothesized that, in the absence of the regulatory effects of estrogen on MMP-9 activity, older women on the AD continuum would exhibit worse biomarker profiles and faster rates of cognitive decline in relation to higher MMP-9 levels, compared to men.

## Methods

### The Alzheimer’s Disease Neuroimaging Initiative (ADNI)

All data used in the preparation of this manuscript were obtained from the Alzheimer’s Disease Neuroimaging Initiative (ADNI) database (adni.loni.usc.edu) in December of 2021. The ADNI is a multi-site longitudinal study aiming to measure the progression of MCI and early AD using multi-modal techniques. For more details regarding the ADNI procedures and diagnostic criteria, refer to www.adni-info.org. Briefly, participants with Mini-Mental Status Examination (MMSE) scores between 24 and 30 (inclusive) and a Clinical Dementia Rating (CDR) of 0, who show no evidence of cognitive impairment or depression, are classified as cognitively normal (CN). Individuals who score between 24 and 30 (inclusive) on the MMSE, have a CDR of 0.5 and objective memory impairment as indicated by education-adjusted Wechsler Memory Scale Logical Memory II performance, and do not have significant levels of impairment in other domains of cognition, essentially preserved levels of daily function and do not meet the diagnostic criteria for dementia, are diagnosed with MCI. Finally, participants with objective memory dysfunction, MMSE scores between 20 and 26 (inclusive), and a CDR ≥ 0.5 who meet the NINDS/ADRDA criteria for probable AD are diagnosed with AD.

### Participants

We included all ADNI participants who had baseline data on plasma MMP-9 and TIMP-1, as well as CSF total tau (t-tau) and phosphorylated_181_ tau (p-tau181), and CSF Aβ_42_ levels below an established cut-point for positivity. We limited our sample to Aβ-positive individuals to ensure that our results are reflective of AD-related pathophysiological processes. MMP-9, TIMP-1, and CSF biomarker data were matched with participants’ clinical diagnostic and medical history data at the same study visit to determine their clinical diagnosis and self-reported presence of cardiovascular disease risk factors including hypertension, smoking, and diabetes.

### Plasma MMP-9 and TIMP-1

Plasma MMP-9 and TIMP-1 measures were obtained from the “Biomarkers Consortium Plasma Proteomics Project RBM Multiplex Data and Primer” file which was downloaded from the ADNI database (adni.loni.usc.edu). Plasma samples were processed on the Luminex Multi-Analyte Profiling (xMAP) platform (Myriad Rules Based Medicine; RBM, Austin, TX). We analyzed two measures of plasma MMP-9 that are available in the ADNI RBM dataset, namely total MMP-9 and free MMP-9 concentration. Total MMP-9 concentration is measured with an assay that allows polyclonal detection of free MMP-9 and bound MMP-9 (i.e., complexes that MMP-9 forms with other compounds such as other MMP species and TIMPs). Free MMP-9 concentration is measured with an assay that uses a monoclonal detection recognizing a single MMP-9 epitope.

### CSF biomarkers

Baseline measures of CSF Aβ_42_, t-tau, and p-tau181 concentrations were obtained from the UPENNBIOMK_MASTER_data.csv file which is publicly available on the ADNI database (adni.loni.usc.edu). ADNI CSF samples were processed with the INNO-BIA AlzBio3 Research Use Only (RUO) immunoassay (Fujirebio, Ghent, Belgium) on the Luminex xMAP platform (Myriad RBM, Austin, TX) according to established protocols [34]. CSF Aβ_42_ concentration was used to determine Aβ status at baseline using a validated ADNI cutoff value of <192 pg/mL [35].

### APOE genotyping

*APOE* genotype data was obtained from the ADNI database (adni.loni.usc.edu). Details regarding blood sample collection and genotyping procedures for ADNI can be found at http://adni.loni.usc.edu/methods. Briefly, *APOE* genotyping is performed on a 3-mL aliquot of EDTA blood using polymerase chain reaction amplification and HhaI restriction enzyme digestion. The samples are then resolved on 4% Metaphor Gel and the results are visualized using ethidium bromide staining [36]. Homozygotes and heterozygotes for the ε4 allele were classified as *APOE* ε4 carriers, while others were categorized as *APOE* ε4 non-carriers.

### Cognitive assessments

Cognition was assessed using MMSE scores and Alzheimer’s Disease Assessment Scale-Cognitive Subscale scores (ADAS-cog), which were obtained from the ADNI database (adni.loni.usc.edu). Higher levels of MMP-9 were previously found to correlate with faster declines in performance on the MMSE and ADAS-cog among a sample of *APOE* ε4-positive ADNI participants with MCI due to AD as indexed by the presence of abnormal levels of CSF Aβ_42_ and/or abnormal levels of CSF p-tau181 [23]. Thus, selecting these measures allows us to compare our results to prior relevant findings.

### Statistical analysis

All statistical analyses were conducted using RStudio version 2021.09.0 (University of Auckland, Auckland, New Zealand). We examined sex differences in demographic and clinical characteristics using a *χ*^2^ test for categorical variables and a Welch’s two-sample *t*-test for continuous variables. Using linear regression models, we tested for an association between sex and free and total MMP-9 levels in the whole cohort and in each diagnostic group. We then used linear regression models testing for an interaction between sex and plasma MMP-9 levels (separate linear regressions for total and free plasma MMP-9) on CSF Aβ_42_, t-tau, and p-tau181 concentrations. Interaction models were fully factorial as they included terms accounting for the main effect of sex and MMP-9. Base models included the covariates of age, *APOE* ε4 status, and plasma TIMP-1 concentration. Fully adjusted models covaried for additional demographic, health, and lifestyle factors that could influence CSF biomarker concentrations of MMP-9 levels, including years of education and cardiovascular risk factors, such as hypertension, diabetes, and smoking. In the case of a significant interaction between total or free plasma MMP-9 and sex, we used the *multcomp* package to simultaneously derive coefficient estimates of the main effect of total or free plasma MMP-9 for men and women from the interaction model.

We used linear mixed effects (LME) models with random intercepts and random slopes to examine sex differences in the association between baseline MMP-9 levels and annual change in MMSE and ADAS-cog scores by testing for a three-way interaction between sex, baseline total or free plasma MMP-9, and time. Base LME models adjusted for age at baseline, *APOE* ε4 status, years of education, and plasma TIMP-1 levels, and fully adjusted models included additional covariates for hypertension, diabetes, and smoking status.

Linear regression and LME models were repeated in groups stratified by diagnosis. Evidence of differences in plasma MMP-9 levels [15], as well as cognitive and AD biomarker profiles between MCI and AD dementia patients [37], provided a robust rationale for diagnosis-stratified analyses of the relationship between plasma MMP-9 levels and CSF biomarker concentrations and rates of cognitive decline, despite the possible loss in statistical power resulting from the smaller size of diagnosis-stratified samples. *P* values < 0.05 were regarded as significant.

## Results

### Participants

Out of the 566 ADNI participants who had available baseline measures of MMP-9 and TIMP-1 concentration, 356 also had measures of CSF Aβ_42_, t-tau, and p-tau181 at baseline. Out of 356 participants with MMP-9, TIMP-1, and CSF biomarker data at baseline, 240 subjects were Aβ positive (CSF Aβ_42_ < 192 pg/mL). The sample of 240 participants who met our inclusion criteria consisted of one CN individual, 146 MCI patients, and 93 AD dementia patients. Given that a separate analysis in the CN group would not be possible with just one participant under that diagnostic category, that subject was excluded, leaving us with a final study sample of 239 Aβ-positive participants. According to the Biomarkers Consortium Project Team who managed the RBM dataset, one participant’s TIMP-1 concentration was determined to be an outlier and was excluded from all subsequent analyses. Three participants with free plasma MMP-9 concentration values smaller than five standard deviations below the sample mean were excluded from analyses of free plasma MMP-9, and one participant with a CSF t-tau concentration greater than five standard deviations above the sample mean was excluded from analyses of CSF t-tau.

Our final study sample of 238 Aβ-positive ADNI participants was predominantly non-Hispanic White (97.5%), had a mean ± SD age of 74.6 ± 7.3 years and 15.5 ± 3.1 years of education, and consisted of 89 (37.4%) women, 145 individuals with MCI, and 93 AD dementia patients. Out of 238 participants in our final study sample, 232 had baseline and at least one follow-up MMSE and ADAS-cog assessment. Sample characteristics are shown in Table 1. Women were younger and had fewer years of education and higher levels of CSF t-tau relative to men (*p*s < 0.01).

**Table 1:** Participant demographic and clinical characteristics and differences by sex

Variable		Men (*N*=149)	Women (*N*=89)	Total (*N*=238)	*p* value
Age (years)		75.69 (7.05)	72.59 (7.40)	74.53 (7.33)	**0.001**
Diagnosis	Mild cognitive impairment	95 (63.8%)	50 (56.2%)	145 (60.9%)	0.246
	Alzheimer’s disease dementia	54 (36.2%)	39 (43.8%)	93 (39.1%)	
*APOE* (ε4-carrier)		99 (66.4%)	64 (71.9%)	163 (68.5%)	0.380
Education (years)		16.0 (3.09)	14.65 (2.92)	15.50 (3.09)	**0.001**
Race	Asian	2 (1.3%)	0	2 (0.8%)	0.480
	Black or African American	2 (1.3%)	2 (2.2%)	4 (1.7%)	
	White	145 (97.3%)	87 (97.8%)	232 (97.5%)	
Diabetes		10 (6.7%)	2 (2.3%)	12 (5.1%)	0.132
Smoking		2 (1.3%)	2 (2.3%)	4 (1.7%)	0.591
Hypertension		96 (64.4%)	55 (62.5%)	151 (63.7%)	0.765
MMSE score		25.58 (2.59)	25.26 (2.24)	25.46 (2.46)	0.325
Plasma total MMP-9		2.36 (0.20)	2.36 (0.21)	2.36 (0.20)	0.898
Plasma free MMP-9		2.15 (0.24)	2.14 (0.23)	2.14 (0.23)	0.743
Plasma TIMP-1		2.04 (0.11)	2.02 (0.10)	2.03 (0.11)	0.124
CSF t-tau (pg/mL)		111.87 (55.3)	134.86 (68.58)	120.42 (61.45)	**0.005**
CSF p-tau181 (pg/mL)		41.32 (19.20)	42.75 (17.13)	41.86 (18.43)	0.563
CSF Aβ_42_ (pg/mL)		134.48 (26.94)	134.62 (25.86)	134.53 (26.49)	0.968

Bold indicates significance (*p*<0.05)

Base and fully adjusted models revealed no sex differences in free or total plasma MMP-9 levels in the entire cohort and in diagnostic groups. We, therefore, sought to replicate prior work demonstrating higher MMP-9 levels in AD dementia patients compared to individuals with MCI [15]. Adjusting for sex, age, *APOE* ε4 status, and plasma TIMP-1 levels, we found that AD dementia patients had significantly higher levels of total plasma MMP-9 than MCI individuals (*β*=−0.06; 95% CI, −0.11 to −0.01; *p*=0.031); however, this difference was attenuated in the fully adjusted model (*β*=−0.05; 95% CI, −0.11 to 0.00; *p*=0.053). Free plasma MMP-9 levels did not differ between MCI and AD dementia patients, regardless of sex.

### Interactive effect of sex and plasma MMP-9 on AD biomarkers

Sex × plasma MMP-9 interactions on CSF AD biomarkers in the whole cohort and in each diagnostic group are summarized in Table 2.

**Table 2:** Summary of sex × plasma MMP-9 interaction effects on AD biomarkers in the whole cohort and in diagnosis-stratified analyses

Interaction between sex and plasma MMP-9 on CSF t-tau						
	Whole cohort		MCI		AD	
	*β* (95% CI)	*p* value	*β* (95% CI)	*p* value	*β* (95% CI)	*p* value
Sex × free MMP-9	98.61 (33.61 to 163.62)	**0.003**	64.03 (−19.49 to 147.55)	0.1	163.81 (49.67 to 277.95)	**0.005**
Sex × total MMP-9	128.68 (55.37 to 201.99)	**<0.001**	119.04 (32.09 to 205.99)	**0.008**	111.33 (−46.47 to 269.13)	0.2
Interaction between sex and plasma MMP-9 on CSF p-tau181						
	Whole cohort		MCI		AD	
	*β* (95% CI)	*p* value	*β* (95% CI)	*p* value	*β* (95% CI)	*p* value
Sex × free MMP-9	21.70 (0.65 to 42.76)	**0.043**	16.25 (−10.46 to 42.96)	0.2	34.27 (−1.91 to 70.46)	0.06
Sex × total MMP-9	21.49 (−3.25 to 46.24)	0.09	28.63 (0.54 to 56.71)	**0.046**	−4.40 (−57.66 to 48.86)	0.87
Interaction between sex and plasma MMP-9 on CSF Aβ_42_						
	Whole cohort		MCI		AD	
	*β* (95% CI)	*p* value	*β* (95% CI)	*p* value	*β* (95% CI)	*p* value
Sex × free MMP-9	−24.10 (−55.01 to 6.81)	0.1	−44.66 (−85.00 to −4.32)	**0.03**	15.39 (−36.91 to 67.70)	0.6
Sex × total MMP-9	−11.00 (−46.16 to 24.15)	0.5	−36.66 (−79.55 to 6.22)	0.09	42.49 (−27.17 to 112.14)	0.2

### Interactive effect of sex and plasma MMP-9 on CSF t-tau

**Fig. 1:**
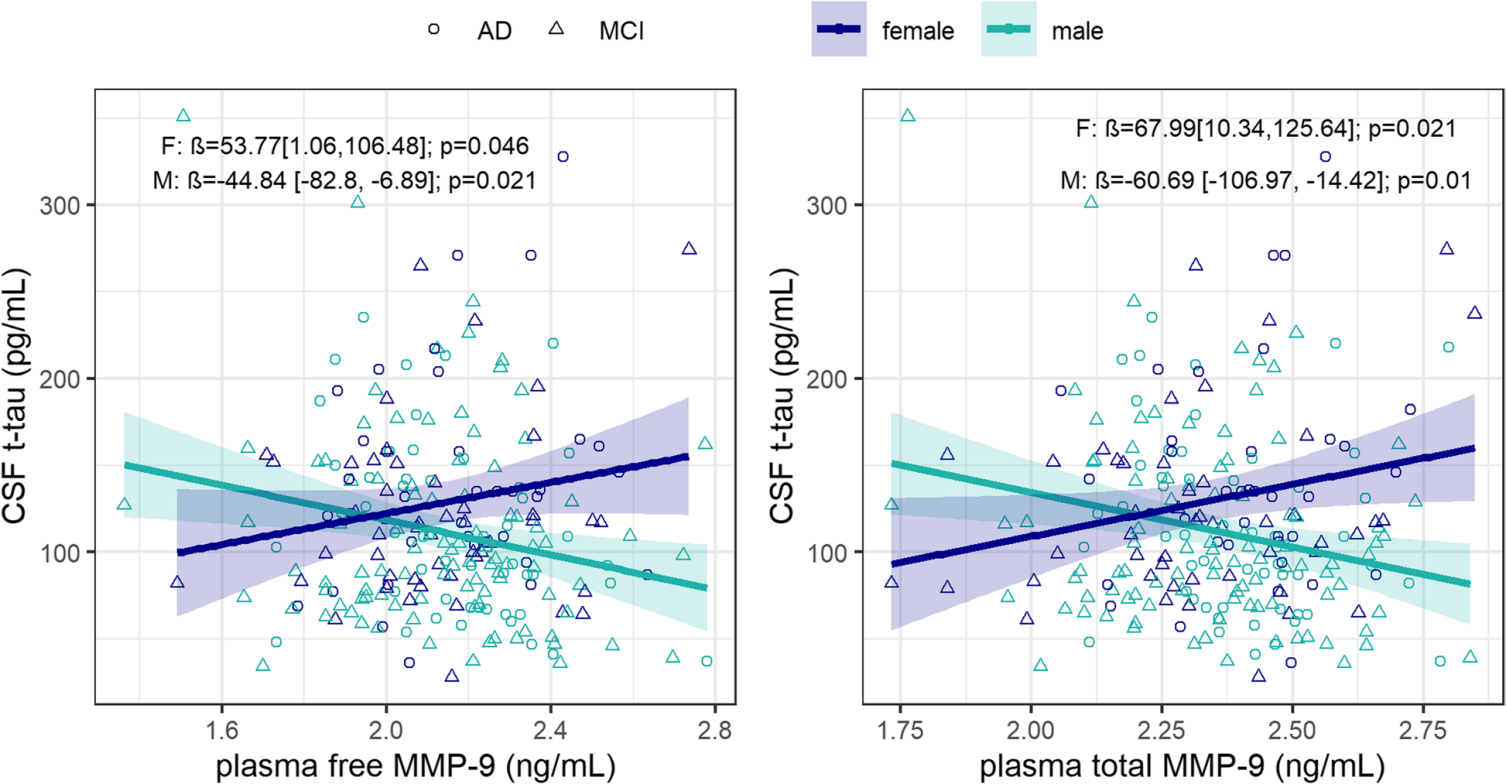
Sex modified the association between free (left) and total (right) plasma MMP-9 levels and CSF t-tau concentration. Higher free and total plasma MMP-9 levels correlated with higher CSF t-tau levels for women (blue), while higher free and total plasma MMP-9 levels were associated with lower CSF t-tau levels for men (green)

There was a significant interaction between sex and plasma free MMP-9 on CSF t-tau levels (*β*=95.96; 95% CI, 31.66 to 160.27; *p*=0.004), which remained significant in the fully adjusted model (*β*=98.61; 95% CI, 33.61 to 163.62; *p*=0.003) (Fig. 1). Higher plasma free MMP-9 correlated with higher CSF t-tau levels among women (*β*=53.77; 95% CI, 1.06 to 106.48; *p*=0.046) and lower CSF t-tau levels among men (*β*=−44.84; 95% CI, −82.80 to −6.89; *p*=0.021). Diagnosis-stratified analyses of fully adjusted models revealed that this interaction was driven by AD dementia patients (*β*=163.81; 95% CI, 49.67 to 277.95; *p*=0.005), as it was not significant in the MCI group. We observed a similar interaction between sex and total plasma MMP-9 levels on CSF t-tau (*β*=121.29; 95% CI, 47.79 to 194.80; *p*=0.001), which persisted after further adjustment for additional covariates (*β*=128.68; 95% CI, 55.37 to 201.99; *p*<0.001). Higher plasma total MMP-9 levels were associated with higher CSF t-tau levels among women (*β*=67.99; 95% CI, 10.34 to 125.64; *p*=0.021) and lower CSF t-tau levels among men (*β*=−60.69; 95% CI, −106.97 to −14.42; *p*=0.010). This modulating effect of sex was driven by individuals with MCI (*β*=119.04; 95% CI, 32.09 to 205.99; *p*=0.008) and was not evident in AD dementia patients.

### Interactive effect of sex and plasma MMP-9 on CSF p-tau181

**Fig. 2:**
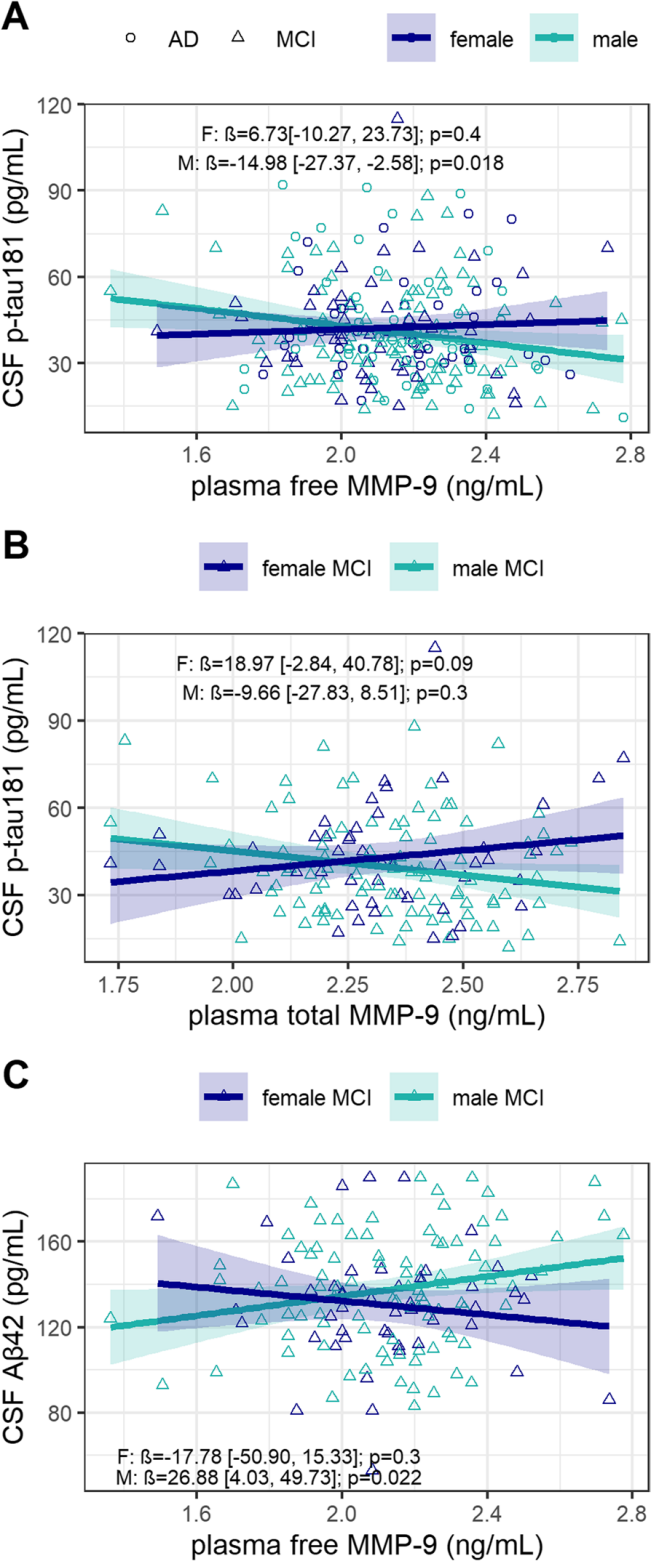
Sex modified the association between plasma MMP-9 and CSF p-tau181 and Aβ_42_. **A** Higher free plasma MMP-9 levels correlated with lower CSF p-tau181 levels for men (green), but there was no association between free MMP-9 and CSF p-tau181 levels among women (blue). **B** There was a trend for a significant correlation between higher total MMP-9 and higher CSF p-tau181 levels among women with MCI, but there was no association in men with MCI. **C** Higher free plasma MMP-9 levels were associated with higher CSF Aβ_42_ among men with MCI (green) but there was no correlation among women with MCI (blue)

There was a trend for a significant interaction between sex and plasma free MMP-9 levels on CSF p-tau181 (*β*=19.92; 95% CI, −0.66 to 40.50; *p*=0.06), which became significant in the fully adjusted model (*β*=21.70; 95% CI, 0.65 to 42.76; *p*=0.043) (Fig. 2A). Higher plasma free MMP-9 correlated with lower CSF p-tau181 in men (*β*=−14.98; 95% CI, −27.37 to −2.58; *p*=0.018), but there was no correlation among women (*β*=6.73; 95% CI, −10.27 to 23.73; *p*=0.4). This interaction was not significant within each diagnostic group. There was no interaction between sex and plasma total MMP-9 levels on CSF p-tau181 in the whole cohort. However, we observed an interaction effect of sex and total plasma MMP-9 levels on CSF p-tau181 among MCI patients (*β*=31.79; 95% CI, 3.76 to 59.82; *p*=0.027), which remained significant in the fully adjusted model (*β*=28.63; 95% CI, 0.54 to 56.71; *p*=0.046) (Fig. 2B). There was no correlation between total plasma MMP-9 and CSF p-tau181 levels among men (*β*=−9.66; 95% CI, −27.83 to 8.51; *p*=0.3), and there was a trend for a correlation between higher total plasma MMP-9 and higher CSF p-tau181 among women (*β*=18.97; 95% CI, −2.84 to 40.78; *p*=0.09). Total plasma MMP-9 levels did not correlate with CSF p-tau181 among AD dementia patients regardless of sex.

### Interactive effect of sex and plasma MMP-9 on CSF Aβ_42_

Although we observed no sex × free plasma MMP-9 interaction on CSF Aβ_42_ in the whole cohort, we found a significant interaction between sex and free plasma MMP-9 on CSF Aβ_42_ in the MCI group (*β*=-41.3; 95% CI, −80.59 to −2.01; *p*=0.040), which remained significant in the fully adjusted model (*β*=−44.66; 95% CI, −85.00 to −4.32; *p*=0.03) (Fig. 2C). Higher free plasma MMP-9 levels were associated with higher CSF Aβ_42_ (reflecting lower Aβ neuropathology) among men with MCI (*β*=26.88; 95% CI, 4.03 to 49.73, *p*=0.022) but there was no correlation among women with MCI (*β*=−17.78; 95% CI, −50.90 to 15.33, *p*=0.3). Free plasma MMP-9 did not correlate with CSF Aβ_42_ in AD, and total plasma MMP-9 was not associated with CSF Aβ_42_ in either diagnostic group, regardless of sex.

### Sex differences in the association between baseline MMP-9 levels and cognition

**Fig. 3:**
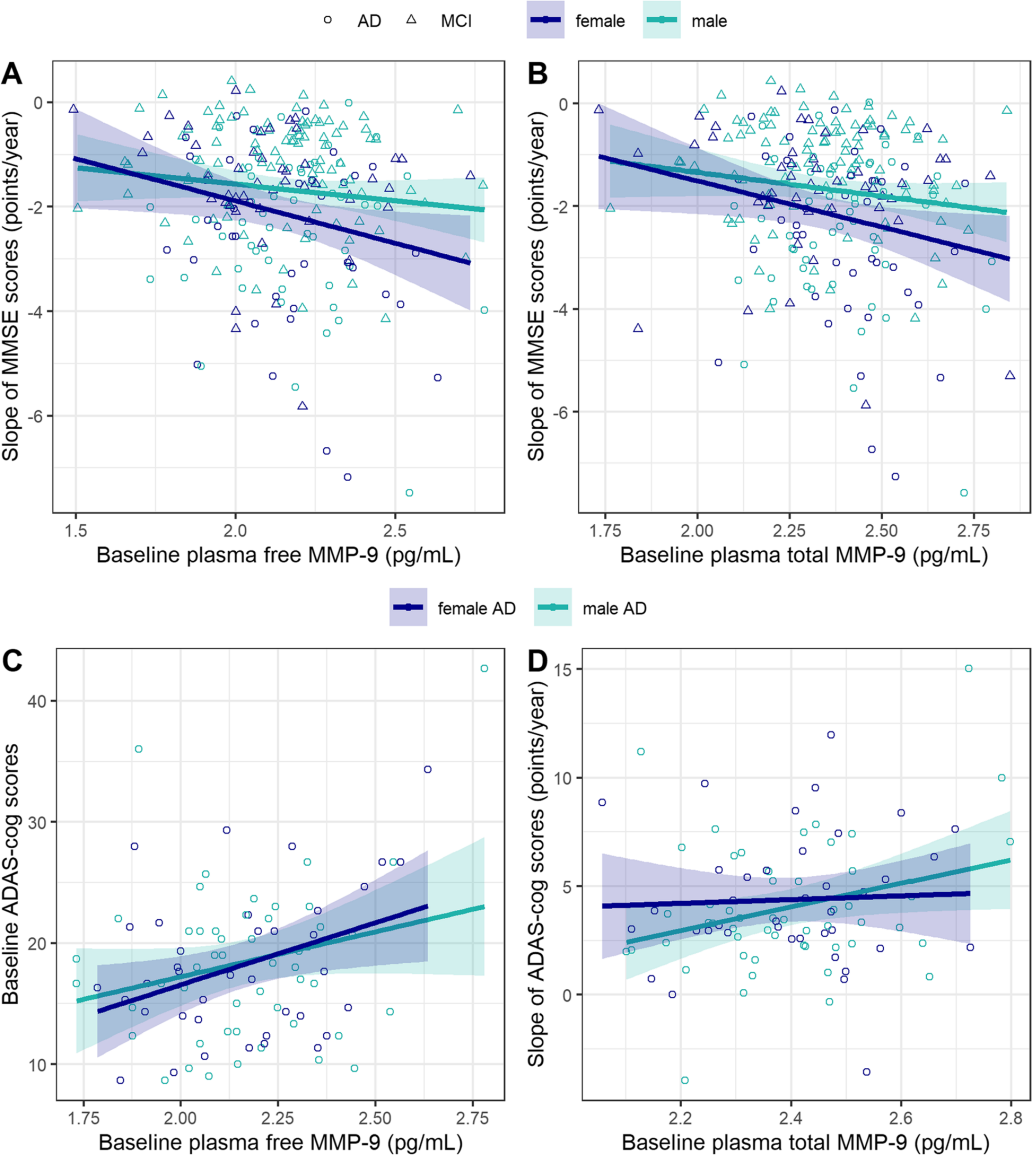
Scatter plots depicting the association between baseline plasma MMP-9 levels and cognitive performance in men and women. **A** Higher levels of plasma free MMP-9 at baseline were associated with a faster decline in MMSE scores in women (blue) but not men (green). **B** Similarly, higher total MMP-9 at baseline correlated with a faster decline in MMSE scores among women but not men. Higher baseline free MMP-9 levels correlated with higher ADAS-cog scores cross-sectionally in women (blue), but not in men (green). **C** Higher baseline free MMP-9 levels correlated with higher ADAS-cog scores cross-sectionally in women with AD dementia, but not in men (green). **D** Higher total MMP-9 levels at baseline correlated with a faster increase in ADAS-cog scores for men with AD dementia, but not for women

There was no significant three-way interaction between sex, baseline MMP-9 levels (free or total), and time on longitudinal MMSE and ADAS-cog scores, which may have been due to low statistical power [38]. Therefore, we stratified our sample by sex to examine the effect of baseline total and free MMP-9 levels on change in MMSE and ADAS-cog scores over time in men and women separately. We observed no cross-sectional association between free or total plasma MMP-9 levels and baseline MMSE scores, regardless of sex and diagnosis. A significant free MMP-9 × time interaction in women (*β*=−2.10; 95% CI, −3.97 to −0.27; *p*=0.027) but not men (*β*=−0.90; 95% CI, −2.16 to 0.38; *p*=0.2) indicated that higher levels of plasma free MMP-9 at baseline were associated with a faster decline in MMSE scores in women (Fig. 3A) after adjusting for age at baseline, *APOE* ε4 status, years of education, and plasma TIMP-1 levels. The effect of baseline plasma free MMP-9 levels on the decline in MMSE scores among women remained significant (*β*=−2.12; 95% CI, −4.02 to −0.25; *p*=0.028) after further adjustment for hypertension, diabetes, and smoking. Diagnosis-stratified analyses revealed that this effect was not significant in either of the stratified groups, likely due to the smaller subsample sizes, but there was a trend for a faster decline in MMSE scores in relation to higher baseline plasma free MMP-9 in women with AD (*β*=−3.77; 95% CI, −7.58 to 0.02; *p*=0.053).

A similar effect was observed for plasma total MMP-9 (Fig. 3B), such that higher total MMP-9 at baseline correlated with a faster decline in MMSE scores among women (*β*=−2.24; 95% CI, −4.32 to −0.18; *p*=0.035) but not men (*β*=−1.05; 95% CI, −2.55 to 0.47; *p*=0.2). This effect among women remained significant in the fully adjusted model (*β*=−2.27; 95% CI, −4.40 to −0.17; *p*=0.036), but was not significant when examined separately in women with MCI and women with AD.

While there was no cross-sectional association between free MMP-9 levels and ADAS-cog scores at baseline in the overall sample, diagnosis-stratified analyses revealed a significant cross-sectional association between higher baseline free MMP-9 levels and higher baseline ADAS-cog scores (indicative of worse performance) (*β*=12.34; 95% CI, 3.02 to 21.65; *p*=0.011) in women with AD (Fig. 3C). Notably, this association was not significant in men with AD (*β*=7.82; 95% CI, −0.87 to 16.57; *p*=0.08). The cross-sectional association between free MMP-9 levels and ADAS-cog scores in female AD dementia patients remained significant in the fully adjusted model (*β*=11.79; 95% CI, 2.45 to 21.12; *p*=0.015). Baseline plasma free MMP-9 did not influence the rate of change in ADAS-cog scores, regardless of sex and diagnosis.

We found no cross-sectional association between baseline total MMP-9 levels and ADAS-cog scores in the whole cohort, regardless of sex. However, diagnosis-stratified analyses showed that among female AD dementia patients, higher baseline total MMP-9 levels correlated with higher baseline ADAS-cog scores (*β*=15.88; 95% CI, 3.71 to 28.1; *p*=0.013) and this effect remained significant in the fully adjusted model (*β*=15.14; 95% CI, 2.33 to 28.00; *p*=0.023). Notably, this association was not significant in male AD dementia patients (*β*=9.53; 95% CI, −1.51 to 20.61; *p*=0.09).

When examining the whole cohort, baseline total MMP-9 levels did not relate to the rate of change in ADAS-cog scores. However, among AD dementia patients, higher total MMP-9 levels at baseline correlated with a faster increase in ADAS-cog scores (indicative of worsening performance) for men (*β*=8.98; 95% CI, 0.27 to 17.68; *p*=0.042), but not for women (*β*=−2.31; 95% CI, −13.39 to 8.52; *p*=0.7) (Fig. 3D) and this effect in men remained significant after further adjustment for cardiovascular risk factors (*β*=9.10; 95% CI, 0.40 to 17.79; *p*=0.039).

## Discussion

Our study’s goal was to examine sex differences in MMP-9 levels and in the association of plasma MMP-9 with CSF AD biomarkers and cognitive decline in individuals on the AD continuum. In a sample of Aβ-positive participants with MCI or AD dementia, we found significant sex differences in the relationship of plasma MMP-9 with CSF biomarker levels, despite similar plasma MMP-9 concentrations among men and women. Specifically, we found that while higher plasma MMP-9 levels correlated with more advanced levels of CSF t-tau, p-tau181, and Aβ_42_ in women, higher plasma MMP-9 levels were associated with more favorable biomarker profiles in men. Furthermore, we found that higher baseline plasma MMP-9 levels predicted a faster decline in MMSE performance among women but not men. Our analyses revealed that associations between plasma MMP-9 and ADAS-cog scores were restricted to AD dementia patients. We found that higher baseline plasma MMP-9 correlated with worse baseline ADAS-cog scores among female but not male AD dementia patients, but higher baseline MMP-9 levels correlated with deteriorating ADAS-cog scores over time among male but not female AD dementia patients.

Consistent with prior evidence of increased plasma MMP-9 levels in AD dementia patients compared to individuals with MCI [13–15], we found higher total plasma MMP-9 concentrations in AD patients compared to participants with MCI. However, we found no sex differences in plasma MMP-9 levels regardless of diagnosis. Estrogen treatment has been shown to inhibit MMP-9 expression and activity in human macrophages and rat microglia [29, 30] and reduce plasma MMP-9 levels in post-menopausal women [32]. In a sample of healthy adults with a mean age of 40.3 years, Kusnierova et al. found lower plasma MMP-9 levels among women, compared to men [39]. These prior findings together with our current observations suggest that the sex difference in plasma MMP-9 levels among health older adults may be driven by pre-menopausal women due to the regulatory effects of estrogen on MMP-9 expression. In contrast, this sex difference in plasma MMP-9 levels may no longer be evident when comparing post-menopausal women to age-matched men. Future longitudinal studies examining sex differences in plasma MMP-9 concentration from midlife into older age will be necessary to assess this possible interpretation of our findings.

Our results revealed that in participants with MCI, higher plasma free MMP-9 levels were associated with higher CSF Aβ_42_, reflecting lower levels of Aβ neuropathology, among men but not women. Several studies have demonstrated that MMP-9 promotes Aβ clearance by degrading aggregate Aβ fibrils [6, 17, 40], and 17β-estradiol treatment was shown to enhance MMP-9 catabolism of Aβ_42_ [33]. A comprehensive review by Duarte et al. suggested that the aromatization of testosterone to 17β-estradiol can facilitate estrogen-mediated neuroprotective mechanisms against Aβ neurotoxicity in older men [41]. It is therefore possible that testosterone aromatization may promote 17β-estradiol-mediated pathways of MMP-9 catabolism of Aβ_42_ [42] and contribute to the positive association between plasma MMP-9 and CSF Aβ_42_ levels we observed in men but not women. However, additional research is needed to experimentally probe this hypothesis.

Similar sex differences were observed in the association between plasma MMP-9 and CSF t-tau and p-tau181, suggesting that men exhibit more favorable biomarker profiles in relation to higher MMP-9 levels, than women. Prior work has shown that MMP-9 inefficiently cleaves tau, thereby releasing microtubule-binding domains of tau that facilitate its oligomerization [20, 21]. These findings are consistent with the correlation between higher plasma MMP-9 and higher CSF p-tau181 and t-tau levels observed in women in our study but are at odds with the negative association between plasma MMP-9 and CSF tau biomarkers seen in men. One study demonstrated that the MMP-9 functional promoter polymorphism associated with elevated blood MMP-9 levels correlated with arterial stiffness among older women but not men [43]. After examining the influence of menopausal status on the observed association between the MMP-9 polymorphism and arterial stiffness in women, the authors found that the effect was significant among peri-menopausal and post-menopausal women, but not pre-menopausal women [43]. Considering prior evidence of a strong association between arterial stiffness and tau pathology [44, 45], sex differences in the association between MMP-9 and CSF markers of tau in our study may be mediated by sex-specific MMP-9 effects on arterial stiffness. Furthermore, given the strong, positive association between Aβ burden and tau pathogenesis [46], the negative association between MMP-9 and CSF t-tau and p-tau181 levels among men may also be due to the seemingly protective effect of MMP-9 on Aβ_42_ burden we observed exclusively in men.

Our study also revealed that higher baseline MMP-9 levels predicted a faster decline in MMSE scores over time in women but not men. This finding is consistent with our observation of worse CSF biomarker profiles in relation to higher MMP-9 levels among women, but not men, and suggests that sex differences in the effect of MMP-9 on AD pathology may have important implications for subsequent cognitive trajectories. Prior investigations of the relationship between circulating MMP-9 levels and MMSE performance have been limited and have simply covaried for sex [16, 23]. One study found no association between plasma MMP-9 levels and MMSE scores [16], whereas another group found a faster decline in MMSE performance in *APOE* ε4-positive, MCI individuals with high levels of plasma MMP-9 [23]. While discrepant findings may be due to methodological differences, our study suggests they may also be due to differences in the proportions of men and women in different samples.

Consistent with prior work showing that the ADAS-cog score is not sensitive to subtle cognitive changes associated with the pre-dementia stage [47], we found that MMP-9 associations with ADAS-cog scores were restricted to AD dementia patients. Interestingly, we found that while cross-sectional associations between higher MMP-9 levels and higher ADAS-cog scores were evident only in women, longitudinal associations between higher MMP-9 levels and worsening ADAS-cog scores were observed only among men. Lin et al. found that female ADNI participants with MCI exhibited a faster decline in ADAS-cog performance over an 8-year follow-up, compared to men with MCI [48], suggesting that women may exhibit greater ceiling effects and plateauing in ADAS-cog performance by the time they are diagnosed with dementia, relative to men.

Our study has some limitations. Our sample was predominantly composed of non-Hispanic White participants which limits the generalizability of our findings to other populations. Moreover, hormone replacement therapy and physical activity have been shown to decrease plasma MMP-9 levels in older adults [32, 49], but their effects on MMP-9 levels were not accounted for in the present study, as this data is not available in the ADNI dataset. Furthermore, studies have demonstrated that MMP-9 effects on blood-brain barrier damage depend on *APOE* ε4 [50, 51], which has been shown to have a stronger effect on AD pathology and clinical outcomes in women [52–54]. Although we adjusted for *APOE* ε4 in analyses, we were unable to examine the role of *APOE* ε4 in the observed interactions between sex and MMP-9, as our sample would have likely been underpowered to detect three-way interactions. Therefore, future studies examining the influence of *APOE* ε4 on sex differences in MMP-9 associations with AD pathology and cognitive impairment are warranted. MMP-9 concentration was derived from plasma, as CSF measures of MMP-9 levels were not available. Prior work suggests that MMP-9 in the blood can indirectly promote AD pathogenesis, by damaging tight junction proteins and endothelial cells, making the blood-brain barrier more permeable to blood-derived neurotoxic molecules, leading to a neuroinflammatory cascade that promotes the accumulation of AD pathology [7]. Whether fluctuations in peripheral MMP-9 can directly influence MMP-9 changes in the CNS remains unclear. Therefore, future research examining sex differences in the effects of MMP-9 on AD pathology and clinical progression should analyze plasma and CSF MMP-9 levels in parallel. Finally, our measures of free and total MMP-9 reflect protein levels rather than enzymatic activity. Although evidence suggests that MMP-9 levels are highly correlated with MMP-9 activity [55], future studies should consider using gelatin zymography to measure MMP-9 activity.

## Conclusions

In conclusion, our findings suggest that sex modulates the influence of MMP-9 on AD-related pathology and cognitive impairment and may crucially inform our understanding of the factors determining its role in AD pathological changes. Numerous studies have shown that women exhibit greater AD risk, more extensive tau pathology, and faster clinical progression than men. Although prior evidence converges on the hypothesis that these sex differences may be related to neuroendocrine changes associated with menopause, precise mechanisms associated with this effect are poorly understood. Our results suggest that MMP-9, which has been previously shown to be regulated by estrogen, may have more detrimental effects on AD-related pathological and cognitive changes in older women. If replicated in larger, population-based samples, our findings could help uncover potential mechanisms contributing to women’s elevated susceptibility to AD.

## Data Availability

All data used in the study’s analysis are available in the ADNI database (adni.loni.usc.edu).

## Abbreviations

Aβ: Amyloid-β
AD: Alzheimer’s disease
ADNI: Alzheimer’s Disease Neuroimaging Initiative
CSF: Cerebrospinal fluid
t-tau: Total tau
p-tau181: Phosphorylated tau at threonine 181
MCI: Mild cognitive impairment
MMP-9: Matrix metalloproteinase-9
MMSE: Mini-Mental Status Examination
ADAS-cog: Alzheimer’s Disease Assessment Scale-Cognitive Subscale
